# A Systematic Review of AI-Driven Prediction of Fabric Properties and Handfeel

**DOI:** 10.3390/ma17205009

**Published:** 2024-10-13

**Authors:** Yi-Fan Tu, Mei-Ying Kwan, Kit-Lun Yick

**Affiliations:** School of Fashion and Textiles, The Hong Kong Polytechnic University, Hong Kong, China

**Keywords:** fabric handfeel prediction, AI in textiles, textile property prediction, tactile simulation

## Abstract

Artificial intelligence (AI) is revolutionizing the textile industry by improving the prediction of fabric properties and handfeel, which are essential for assessing textile quality and performance. However, the practical application and translation of AI-predicted results into real-world textile production remain unclear, posing challenges for widespread adoption. This paper systematically reviews AI-driven techniques for predicting these characteristics by focusing on model mechanisms, dataset diversity, and prediction accuracy. Among 899 papers initially identified, 39 were selected for in-depth analysis through both bibliometric and content analysis. The review categorizes and evaluates various AI approaches, including machine learning, deep learning, and hybrid models, across different types of fabric. Despite significant advances, challenges remain, such as ensuring model generalization and managing complex fabric behavior. Future research should focus on developing more robust models, integrating sustainability, and refining feature extraction techniques. This review highlights the critical gaps in the literature and provides practical insights to enhance AI-driven prediction of fabric properties, thus guiding future textile innovations.

## 1. Introduction

With the rapid technological advancements of the Fourth Industrial Revolution, the application of artificial intelligence (AI) in the textile industry is becoming a forefront area of research and practice. This development builds on a long history of innovation, beginning with the mechanization introduced during the First Industrial Revolution and further propelled by automation and digitalization trends over the past decades. AI has introduced unprecedented innovations in textile design, manufacturing, and quality control, particularly in predicting fabric properties and handfeel.

Fabric properties, such as strength, elasticity, breathability, and handfeel characteristics like softness and roughness, are critical factors that determine the final performance of textiles [1]. Traditionally, the measurement and evaluation of these properties relied heavily on laboratory testing and expert judgment, which are not only time-consuming and costly but also inherently subjective [2,3]. As a result, there has been a growing interest in leveraging AI technology to automate the prediction of these properties, thus driving the industry toward greater efficiency, consistency, and sustainability.

AI-based approaches, particularly machine learning and deep learning models, have shown significant potential in predicting fabric properties and handfeel across multiple dimensions. These models can effectively handle large-scale datasets, capture complex nonlinear relationships between fabric attributes, and provide reliable predictions that, in some cases, surpass the accuracy of conventional methods [4,5]. For instance, AI techniques can incorporate diverse factors such as environmental conditions and user-specific preferences, which are typically overlooked in traditional testing methods. However, despite these advantages, AI models still face several challenges, including limited generalizability due to dataset constraints, high computational requirements, and lower interpretability [6,7]. These limitations may hinder their broader adoption in real-world applications that require transparent decision-making and explainability [5,7].

Compared to traditional methods, such as the Kawabata Evaluation System (KES) and Fabric Assurance by Simple Testing (FAST), which rely on physical measurements, AI models can handle complex, multidimensional data and provide real-time feedback, making them highly suitable for the fast-paced textile industry [8,9]. While conventional methods often struggle to address the nonlinear relationships between various fabric properties, AI excels in identifying hidden patterns and offers real-time optimization, which is critical for meeting consumer demands and accelerating product development [10].

Most existing studies focus on predicting a single property or handfeel characteristics, with little attention given to a systematic literature review (SLR) of AI technologies that are used to predict more than one fabric property and handfeel itself. Furthermore, as the industry shifts toward more sustainable practices, there is a need to develop AI models that not only optimize fabric performance but also account for environmental impact and resource efficiency [7,11]. Moreover, AI-driven innovations can contribute to new business models and profit streams by introducing advanced capabilities such as on-demand customization and intelligent supply chain management. Therefore, a comprehensive review of AI-driven techniques to predict fabric properties and handfeel is not only essential for mapping out current research but also to provide critical insights into future research directions.

This study presents a systematic review of AI-driven techniques to predict fabric properties and handfeel by focusing on the mechanisms of these models, the diversity and scale of the datasets, and the accuracy and practical effectiveness of the predictions. We will review successful cases in current research, analyze their applications and limitations, and explore ways to enhance model accuracy in various application scenarios. Additionally, this paper will discuss the potential applications of these technologies in environmental sustainability and future textile developments.

Following this introduction, Section 2 will discuss the application mechanisms of AI models in predicting fabric properties. Section 3 will review the current research, and focus on the performance of different models and their real-world applications. Section 4 will analyze the impact of dataset diversity and scale on the accuracy of model predictions. Finally, Section 5 will outline future research challenges and directions to promote the broader application of AI in the textile industry.

## 2. Materials and Methods

**Figure 1:**
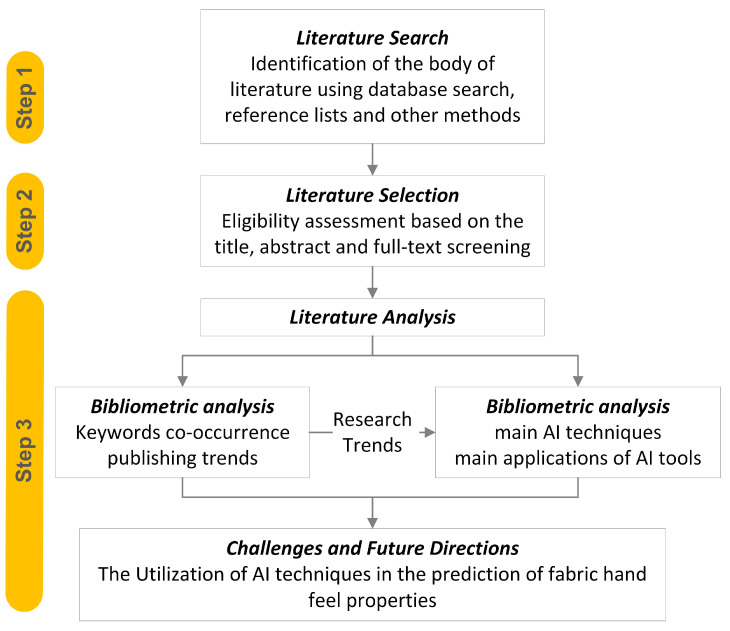
Visual flow of research process.

This study reviews the effectiveness of AI in predicting handfeel-related fabric properties, such as softness, stiffness, and drape. This method was chosen because it provides a systematic approach that ensures objectivity, rigor, and transparency while offering insights into theoretical knowledge and current trends and developments relevant to the research question [12]. We followed the PRISMA 2020 guidelines (Registered on OSF Registries: accessed 19 September 2024, https://doi.org/10.17605/OSF.IO/CYD9P) to ensure transparency and rigor in the selection, analysis, and synthesis of studies. We conducted a focused search on the Web of Science (WoS) database, and targeted studies with successful predictions of fabric properties. The review identified research work with a high accuracy (over 80%) in predicting attributes like tensile strength and elasticity by using datasets with a few dozen to several hundred fabric samples. In addition to the database searches, manual and reference list searches were conducted to identify additional papers. After the selection process, the identified studies were further analyzed by using quantitative and qualitative methods to help answer the primary research questions, as shown in Figure 1.

### 2.1. Eligibility Criteria

The use of AI in measuring and predicting fabric properties is extensive, which complicates the identification of relevant studies on handfeel. To refine the literature search, we focused on three key categories: (1) handfeel measurement and evaluation systems, (2) fabric properties and predictive features, and (3) AI and machine learning models. These areas are integral to tactile perception research. The search was confined to publications from 2014 to August 2024 to ensure the inclusion of the most recent findings.

**Figure 2:**
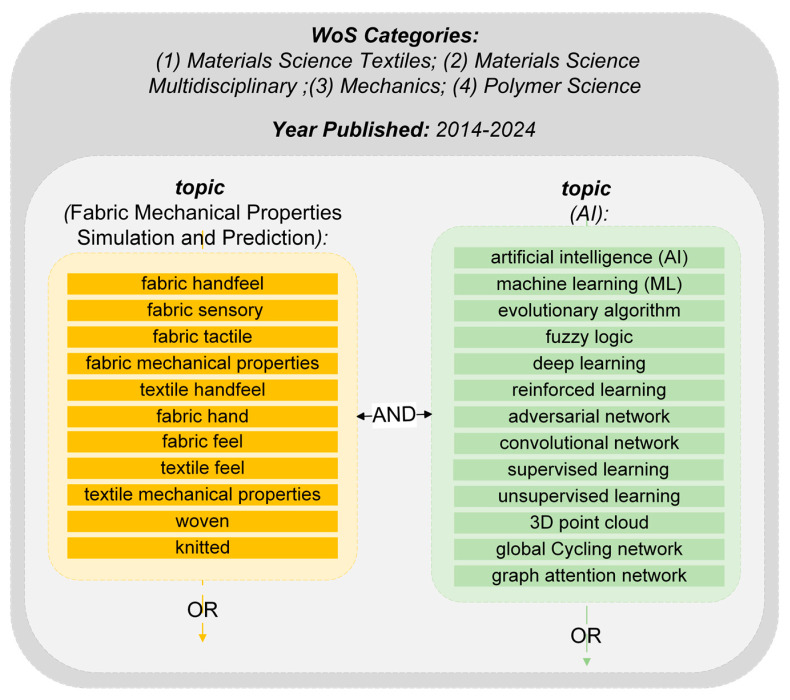
Illustration of the literature search process. The dark grey outer box represents the initial filtering by topic categories and year of publication, followed by the application of specific keywords within these limits (inner box).

**Figure 3:**
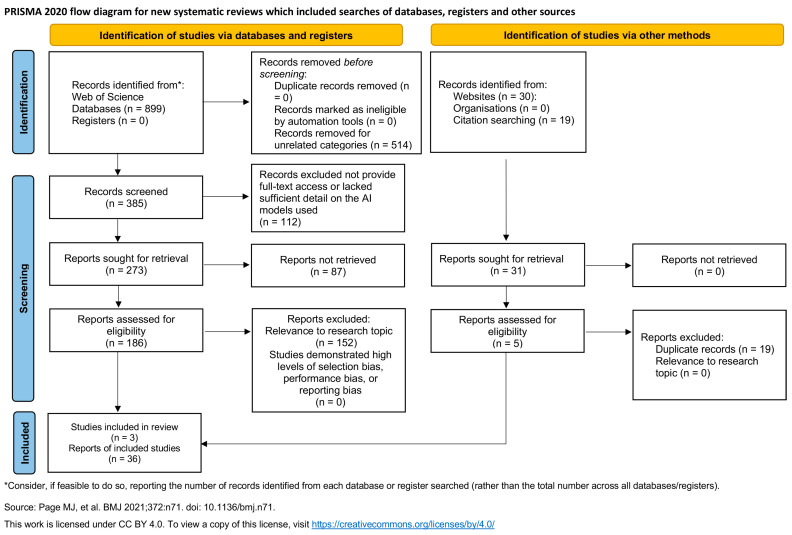
PRISMA flow diagram of literature review [13].

The initial search was conducted on the WoS database, which was chosen for its peer-reviewed content and detailed categorization by research area, which is crucial for finding articles on fabric handfeel. We restricted the search to journal articles and conference proceedings related to Materials Science, Textiles, Computer Science, Software Engineering, and Materials Science Composites, published in English. The keywords were divided into two groups: one that targets AI tools and the other on fabric measurement and prediction terms. The search string included “artificial intelligence”, “machine learning”, “neural network”, and other related terms (Figure 2). As shown in Figure 3, the initial search yielded 899 results from the WoS database that met the search criteria, followed by a rigorous literature selection process. The identified studies were screened according to the inclusion criteria in Table 1.

Data extraction followed a predefined form that captured key study characteristics, including AI models, dataset size, and outcome measures such as prediction accuracy, mean absolute error (MAE), and root mean square error (RMSE). In addition to these primary outcomes, variables such as dataset diversity, preprocessing techniques, and AI model details (algorithms, parameters, and training procedures) were also recorded. Funding sources were noted to assess potential conflicts of interest, while publication year and journal type were used to track research trends. When information was unclear, assumptions were made based on the study context, and any missing data that could not be inferred were noted as study limitations. Two reviewers independently extracted the data, resolving discrepancies through consensus.

Papers that are not related to the categories were excluded, and only English-language papers were retained, which resulted in the elimination of 514 items to provide 386 papers. Title and abstract reviews that lacked sufficient detail on the used AI models eliminated 112 papers to further reduce the number of studies to 273 relevant papers. Due to 87 reports not being retrievable, the number of papers was reduced to 186 for eligibility assessment. A full-text assessment eliminated an additional 152 papers that did not directly contribute to the research question but originated instead in unrelated disciplines like computer science or other material sciences.

Concurrently, an unstructured search that used the same keywords was conducted in different online repositories, Google Scholar, and citation searching to identify additional potentially relevant papers. This approach contributed an additional 49 papers. Full-text screening process was applied to papers collected from the other sources. After the screening, 5 papers met the inclusion criteria. Ultimately, 5 papers from other sources were included in the review, and, in the end, 39 studies were used for the analysis (Figure 3).

### 2.2. Analyzing the Literature

The selected literature sample, which included 39 studies, was analyzed using both quantitative and qualitative methods. The quantitative analysis was conducted to gain insights into the characteristics of the collected sample, focusing on publication trends, citation patterns, and key research topics. This was accomplished using bibliometric techniques, a mathematical analysis of bibliographic units, to systematically examine citation patterns and trends in AI-driven prediction of fabric properties. By analyzing publication data, keywords, and citations, this approach provided a comprehensive view of how AI techniques have been applied in textile research over time and across different subfields.

A three-step research process was employed in this study. Two researchers conducted the literature search and data collection independently based on predefined criteria. A third researcher performed cross-validation to ensure consistency in inclusion/exclusion decisions, keyword extraction, and data accuracy. The third researcher followed specific quality control parameters, such as verifying the consistency of data extraction and resolving disagreements through consensus. This process was aligned with PRISMA guidelines to maintain inter-rater reliability and reduce potential bias.

A qualitative analysis was carried out to further explore the findings from the quantitative data. This approach focused on reviewing AI-driven techniques for predicting fabric properties, such as softness, stiffness, and drape, with particular attention to model mechanisms, dataset diversity, and prediction accuracy. Through this dual approach, the analysis identified key trends, challenges, and limitations, such as the generalizability of AI models and the impact of data variability on model performance, thus providing a detailed understanding of the current state of research in this domain.

## 3. Results and Analysis

### 3.1. Bibliometric Analysis

#### 3.1.1. Publication Trends

**Figure 4:**
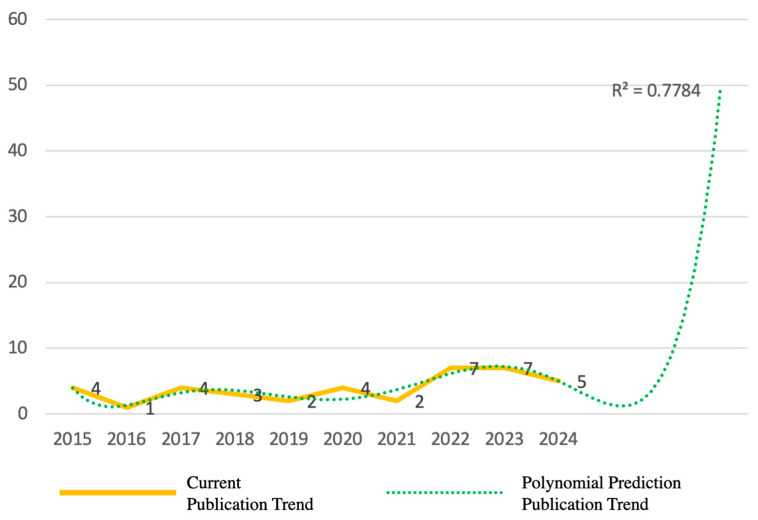
Number of related articles published year by year.

This study focuses on literature from the ten-year period between 2014 and 2024. Notably, there has been a significant increase in relevant publications from 2022 to 2024, with 17 papers (44%) being published during this time. Figure 4 presents an exponential forecasting curve (R^2^ = 0.7784). This trend underscores the novelty of the research area, and emphasizes the need for a comprehensive review. The analysis found that 36 of the 39 papers are journal articles, predominantly published in 21 different journals. The “*Textile Research Journal*” published five articles, which indicates that this journal prioritizes prediction techniques for textile performance. Other journals, like *ACM Transactions on Graphics* and *Indian Journal of Fibre & Textile Research*, also published multiple articles, which reflects a broader interest in AI applications for predicting and evaluating fabric hand.

#### 3.1.2. Keyword Analysis

**Figure 5:**
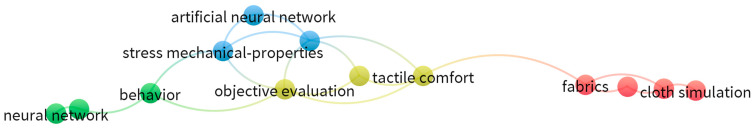
Visual representation of key research topics in AI-driven fabric property prediction using VOSviewer (version 1.6.20). The source data for this visualization were obtained from the Web of Science database.

The keywords were analyzed by using the full counting method, which highlights significant relationships within a dataset. The analysis identified 13 keywords with more than 2 occurrences, to form a visual network that categorizes the research into four clusters (Figure 5). The most frequent keywords are “fabric hand” and “objective evaluation”, which show a strong focus on the quantitative assessment of fabric properties by using AI tools. The clustering revealed three main research themes: “AI-driven evaluation and prediction for handfeel characteristics”, which focuses on tactile comfort and objective evaluations; “AI-driven evaluation and prediction for predicting fabric mechanical properties”, which emphasizes the mechanical properties and performance; and “AI-driven predictive modeling for fabric drape”, which centers on fabric drape and simulation processes.

Based on the keyword co-occurrence in the analyzed sample, two methods for the network visualization were considered: (1) automatically extracting frequently used terms from titles or abstracts, and (2) generating data maps by using directory metadata keywords [14]. This study employs the second method to clearly identify the primary research themes by using predefined keywords that align with the main topics. The bibliographic metadata was gathered by using EndNote (version 21.0.0.19023) and analyzed with VOSviewer.

### 3.2. Content Analysis

#### 3.2.1. Measurement and Evaluation Systems

- Objective and Subjective Evaluations of Fabric Properties

The evaluation of fabric properties and handfeel is critical in textile research, which encompasses both subjective and objective methods. The Kawabata Evaluation System (KES) is a key tool for measuring mechanical properties related to fabric handfeel, including bending, shear, tensile, and compression stiffness, along with surface smoothness and friction [15]. Other systems like Fabric Assurance by Simple Testing (FAST) [16] and the Fabric Touch Tester [17] provide objective evaluations as well. The Fabric Touch Tester, for instance, assesses compression, surface friction, thermal, and bending properties to offer a comprehensive index of fabric handle characteristics [18].

Recent research has increasingly integrated subjective and objective methods to combine sensory analysis with physical measurements. The authors in [19] highlighted individual differences in tactile perception by analyzing finger sliding over fabrics, using KES to measure physical properties and proposing skin vibration as an alternative measure for fabric handfeel. Additionally, innovative methods like the three-dimensional (3D) drape model, which uses a principal component analysis (PCA), have been explored for the objective assessment of fabric handfeel [20].

- Innovative Methods for Drape Measurement

Recent research has introduced advanced methods to improve the accuracy and applicability of fabric drape measurements. One method uses a reciprocating device to simulate fabric movement to identify key factors like node number, amplitude, and the position of the first node, which are crucial for assessing dynamic drape [21]. Another approach combines multidirectional stiffness and drape measurement into a single method by introducing parameters like projection area and length, which correlate well with bending performance and drape, thus offering a more comprehensive evaluation than traditional methods [22].

A learning-based method that uses a drape tester adopts a model-in-the-loop strategy, which uses regression-based neural networks to estimate the simulation parameters, thus enhancing fabric simulation accuracy [23]. Additionally, 3D-printed human models enable more realistic static and dynamic drape evaluations, which can capture 3D fabric behavior [24]. Methods that use Kinect sensors provide non-contact 3D drape measurements, thus correlating well with traditional and subjective evaluations [25]. The Textechno Drapetest device uses a digital image analysis system and laser triangulation sensor in Christ et al. [26] to measure the drape effects, thus offering insights into controlling fabric behavior during 3D shaping [26]. Lastly, Kim [27] used a method that involves a depth camera with an elevating device to compare traditional drape testing with 3D scanning, thus adding valuable insights into the drape phenomena of fabric through 3D analysis.

#### 3.2.2. Fabric Properties and Predictive Features

In AI-driven techniques to predict fabric properties and handfeel, key attributes like mechanical and sensory properties are vital for accurate modeling and predicting tactile comfort and overall handfeel.

Texture, weave parameters, tactile comfort, surface friction, and functional surface treatments are key factors in AI-driven predictions. Seçkin et al. [7] used machine learning algorithms like XGBoost and random forest (RF) to analyze texture and weave parameters from microscopic images, and created a dataset with 458 inputs and 4 outputs, which led to accurate predictions of fabric properties and consistent product quality. Tadesse et al. [28,29] and Ahirwar and Behera [24] focused on mechanical properties and handfeel attributes, and utilized tools like KES-FB, PCA, artificial neural networks (ANNs), and adaptive network-based fuzzy inference systems (ANFISs) to predict tactile comfort, thus highlighting the importance of both subjective and objective assessments. Surface friction, crucial for comfort, was modeled by Ezazshahabi et al. [30], who used a genetic algorithm to effectively predict tactile characteristics based on structural parameters, which streamlines design processes. Additionally, Tuigong and Xin [31] showed that mechanical and surface properties can be used to predict fabric hand stiffness, thus further highlighting the role of these characteristics in fabric property evaluation. Functional surface treatments, including finishes and coatings, significantly impact fabric handfeel, as shown by Tadesse et al. [28], who used fuzzy logic and neural networks to predict hand values to optimize production for consumer preferences [28]. Thermal physiological properties, vital for comfort, were integrated into handfeel models by Xue et al. [32], thus enhancing the prediction of consumer preferences [32]. Finally, Das and Shanmugaraja [33] predicted weave patterns, which are essential for tactile and aesthetic quality by using ANNs to improve accuracy and efficiency in fabric production.

#### 3.2.3. AI and Machine Learning Models

In AI-driven predictions of fabric properties and handfeel, a number of different AI and machine learning models are used to enhance accuracy and efficiency in the textile industry. Automated machine learning (AutoML) technologies simplify model selection and optimization, as seen in Metin and Bilgin [5], who evaluated seven open-source tools. EvalML, an AutoML library, excels in determining the mean absolute error (MAE), while AutoGluon has the best performance in calculating the mean absolute percentage error (MAPE), root mean square error (RMSE), and R-squared, thus highlighting the need to balance accuracy with computational efficiency in predicting fabric quality [5].

Machine learning algorithms like XGBoost and RF have proven effective in predicting texture and weave parameters. In Seçkin et al. [7], XGBoost achieved a 0.987 accuracy in texture classification, and RF had the lowest MAE in specific mass prediction, which shows the potential of these algorithms in enhancing the accuracy of production processes and ensuring a consistent fabric handfeel.

Deep learning models, including convolutional neural networks (CNNs) and hybrid YOLOv4-R-CNN models, are used to detect fabric detects, and are crucial for maintaining high fabric quality [34,35]. These models also predict material parameters like stiffness and damping, which are essential for accurate fabric simulations in virtual environments. Mao et al. [36] used deep learning models such as Transformer, to obtain a 99.01% accuracy in predicting key material parameters, thereby improving the realism and efficiency of fabric simulations.

ANNs are extensively employed to predict various fabric properties, including thermal characteristics, air permeability, and tensile strength. Jhanji et al. [37] highlighted the effectiveness of ANNs in modeling complex and nonlinear relationships, while Erenler and Oğulata [34] achieved a high correlation (R = 0.99366) in predicting air permeability based on factors like weft count and weave pattern. Kothari and Bhattacharjee [38] applied ANNs to predict thermal properties and capture the intricate relationships between fabric structure and thermal behavior more robustly than traditional methods. Ahirwar and Behera [24] showed a strong correlation (0.82) between ANN predictions and subjective assessments of fabric handfeel, while Elkateb [4] validated the utility of ANNs in predicting mechanical properties like tensile strength and bending stiffness, which underscores their potential to enhance customer satisfaction through accurate predictions of handfeel [4,39].

## 4. Applications

**Figure 6:**
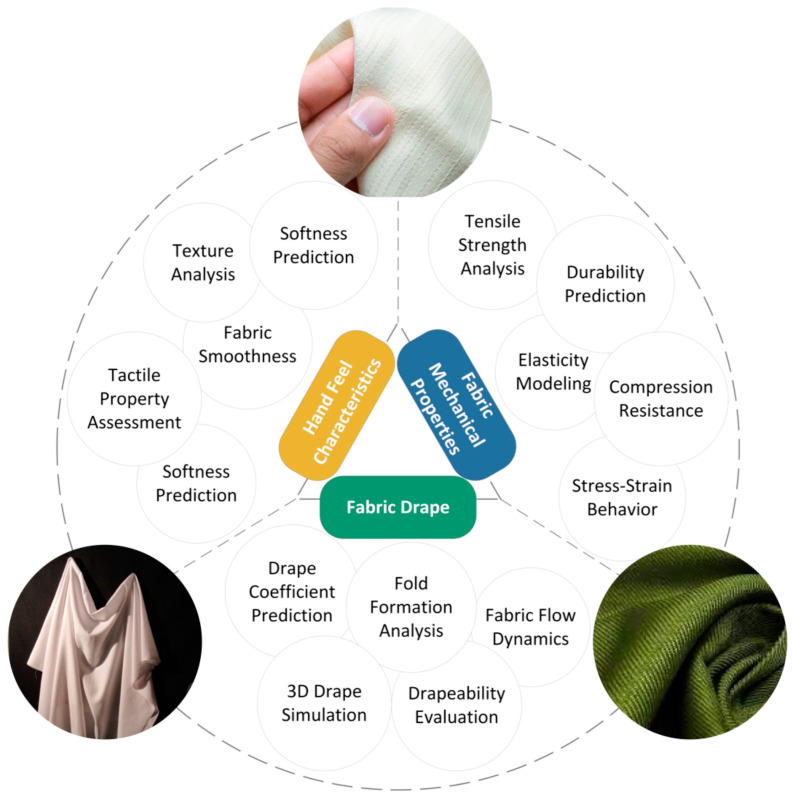
Overview of primary identified uses for AI in predicting fabric handfeel.

The themes identified through the application of AI include (1) predictive modeling for simulation, (2) optimization of fabric properties, and (3) evaluation and classification driven by AI, which are analyzed in depth in this section (Figure 6). Within each theme, the following aspects are reviewed:

1. The application of AI algorithms for specific fabric properties;
2. How AI has overcome challenges in the prediction of fabric properties, particularly with complex datasets;
3. Modifications made to AI algorithms to accommodate specific application domains.

### 4.1. Prediction of Fabric Handfeel Characteristics

AI techniques such as extreme learning machines, genetic algorithms, fuzzy logic, ANFISs, and deep learning have been used in studies to predict various fabric properties with high accuracy. These methods analyze features derived from tactile sensors, visual attributes, and mechanical measurements to predict the tactile qualities of fabrics. The datasets used in these studies vary widely, ranging from a few dozen textile samples to tens of thousands of images, thus reflecting the scalability and adaptability of AI techniques in this field (Table 2).

In the application of AI for predicting fabric handfeel characteristics, various techniques have been employed for different fabric properties. For instance, Rasouli et al. [40] used extreme learning machines to classify textures such as roughness and smoothness, and obtained a 92% rate of accuracy with a dataset of 10 graded textures. Xue et al. [41,42] applied genetic algorithms and ANFISs to predict tactile properties like softness and flexibility, with their models obtaining close to a 95% accuracy in weight distribution and minimal predictive errors on an 11-point scale, respectively. Gültekin et al. [43] utilized deep learning techniques, specifically ResNet-50, to predict the tactile properties based on visual features, and obtained a 99.3% accuracy rate in woven types of fabric and an error level of less than 10% in yarn quality evaluation. Finally, Tadesse et al. [28,29] combined ANNs with fuzzy logic models to predict tactile comfort scores and mechanical properties, with their models showing low error rates and high prediction accuracy.

### 4.2. Prediction of Fabric Mechanical Properties

Fabric mechanical properties such as shear strength, elasticity, bending stiffness, and tensile strength are essential for determining the durability, functionality, and application suitability of textiles. Traditional methods of determining these properties often involve complex physical tests, which can be time-consuming, costly, and sometimes less adaptable to varying conditions. AI techniques are a promising alternative, and allow more efficient, accurate, and scalable predictions of the mechanical behaviors of fabric (Table 3). This approach not only enhances the accuracy of material assessments but also facilitates the development of new textile materials with tailored mechanical characteristics.

The AI-driven prediction of the mechanical properties of fabrics has been used across various types of fabric with notable success. Basit and Luo [44] used a finite element analysis (FEA) along with the Bernoulli–Euler beam theory and Coulomb’s friction model to accurately predict shear force and deformation patterns. Kilic [45] applied neural networks and regression models to predict fabric strength by focusing on bursting and tensile strength, and obtained an R^2^ of 0.765 and close-to-real values with fuzzy logic, thus demonstrating the potential of AI in predicting strength. Ribeiro et al. [46] used automated machine learning and deep neural networks to predict warp and weft elasticities, and obtained a normalized mean absolute error (NMAE) of 4% and 87% accuracy for textile composition, thus underscoring the accuracy of AI in predicting elasticity. Gültekin et al. [43] effectively modeled air permeability and porosity by using ANNs, and they obtained a high regression value (R = 0.99).

In terms of bending and stiffness properties, Feng et al. [23] utilized deep neural networks and in-the-loop simulation, and significantly improved simulation fidelity in predicting bending stiffness, while Koptelov et al. [48] achieved less than 10% error in predicting 3D textile architecture with the use of CNNs and LSTM networks, thus highlighting the capability of AI in handling complex structural properties.

For comprehensive mechanical properties, Kilic et al. [43] showed that there is a strong agreement between experimental and simulated in-plane shear properties by using FEA and analytical methods. Sperl et al. [49] predicted the stiffness, nonlinearity, and anisotropy in knitted fabrics with yarn-level simulation and thin-shell models. They reported an average error of 17.59% for the stretch force and 16.84% for compression. Hsu et al. [50] combined CNNs with FEA to predict woven composite properties with less than 5% error, while Malashin et al. [51] obtained an accuracy of 90.2% with neural networks and 89.9% with support vector machines in predicting tensile strength, compressive and bending strength, and elongation in textile polymer composites. These studies collectively show the efficacy and versatility of AI in predicting the different mechanical properties of fabric with a high degree of accuracy.

### 4.3. Prediction of Fabric Drape

The prediction of fabric drape is a vital aspect of textile engineering, as drape characteristics significantly influence the aesthetic and functional qualities of garments and other textile products. Traditionally, fabric drape has been assessed through physical testing methods, which, while effective, can be labor-intensive and limited in scope. The integration of AI to predict fabric drape has provided new possibilities for more accurate, efficient, and scalable assessments. AI techniques such as fuzzy logic, finite element method (FEM), and CNNs have been employed to predict various drape-related properties, which range from drape coefficients and flexural rigidity to garment fit and tactile sensation, see in Table 4.

The AI applications for predicting fabric drape have shown significant advancements across various studies, with each focusing on different aspects of drape and using different techniques. Kilic [45] employed fuzzy logic combined with image analysis to predict fabric drape coefficients, and obtained an accuracy within 1% of that with the traditional Cusick’s method. This approach shows the potential of AI to closely replicate established physical testing methods. Hamdi et al. [53] also used fuzzy logic to predict drape behavior in woven fabrics, and obtained high correlations across multiple metrics such as drape coefficient and folds depth index, thus further reinforcing the reliability of AI in accurately modeling drape characteristics.

Hübner et al. [52] focused on the drape behavior of 3D woven fabrics by using FEM and a hyperelastic model, and validated their simulations with the experimental results. They highlighted the effectiveness of AI models in simulating complex drape behaviors, which is crucial for advanced textile engineering. In the realm of garment fit and drape characteristics, Oh and Kim [54] applied ANNs in combination with drape simulation, which leads to improved fit scores compared to traditional methods, although specific accuracy rates were not detailed.

Youn et al. [6] expanded the scope by using CNNs with ResNet-18 and self-attention mechanisms to predict both the drape and mechanical properties of fabrics. Their work achieved normalized MAEs that range from 3% to 51%, thus showing the versatility of AI in handling multiple fabric characteristics simultaneously. Finally, Lee et al. [20] integrated fuzzy C-means clustering with ANNs to predict drapability and tactile sensation, particularly softness, with an accuracy of 83.5% across 777 fabric samples. This study underscores the effectiveness of AI in evaluating not only the drape but also the tactile qualities of fabrics.

## 5. Challenges and Future Directions

### 5.1. Challenges and Limitations

Based on the results of the literature review presented in this study, three specific sets of challenges (which correspond to the identified application domains) are summarized: dataset limitations, complex modeling requirements, and hardware constraints.

Dataset Limitations

The generalizability and predictive power of AI models largely depend on the quality and diversity of training datasets. Current datasets used for fabric property prediction tend to focus on specific fabric types or limited mechanical properties, resulting in a lack of representation across the diverse range of textile materials. This absence of comprehensive datasets increases the risk of bias, model overfitting, and reduced performance when the model is applied to unseen data. Additionally, for subjective attributes such as handfeel, the absence of standardized measurement methods further complicates the dataset construction. As a result, these limitations restrict the models’ applicability and diminish the predictive accuracy for more complex fabric properties.

To address these challenges, the development of standardized and high-quality benchmark datasets encompassing a broader range of fabric properties is necessary. Methods such as synthetic data generation through Generative Adversarial Networks (GANs) or physics-based simulations can be considered to augment existing datasets, thereby enhancing data diversity and improving model robustness. Moreover, the integration of subjective sensory evaluations with objective physical measurements would provide a more holistic and comprehensive dataset, enabling AI models to better capture the multifaceted nature of fabric properties.

Complex Modeling Requirements

The inherent complexity and nonlinearity of fabric properties, such as drape, stiffness, and handfeel, present significant challenges for AI models. Simplified assumptions or linear approximations employed in traditional modeling approaches often fail to capture the true underlying dynamics of fabric behaviors, leading to suboptimal predictions. Complex modeling is further challenged by the multifactorial nature of fabric properties, where various parameters such as material composition, weave structure, and environmental factors interact in nonlinear ways. This complexity necessitates the use of advanced modeling techniques capable of representing such intricate relationships.

Addressing these challenges requires the implementation of multi-scale modeling approaches, which can capture interactions at both the microstructural and macroscopic levels of textile properties. Additionally, hybrid models that combine machine learning with physics-based simulations can leverage the strengths of both approaches, providing more accurate and explainable predictions. Techniques such as transfer learning and meta-learning can also be adopted to improve the adaptability of models to new fabric types and properties, reducing dependency on extensive retraining and large datasets.

Hardware and Computational Constraints

The computational demands of AI models, particularly deep learning architectures, pose significant challenges in the context of fabric property prediction. High-dimensional data, complex model structures, and large-scale datasets necessitate substantial memory and processing power, often requiring the use of specialized hardware such as Graphics Processing Units (GPUs) or cloud-based computing resources. This dependency on advanced hardware can limit the accessibility of AI-driven methods to researchers and industry practitioners, particularly those with restricted computational resources.

To mitigate these hardware constraints, model optimization techniques such as pruning, quantization, and knowledge distillation should be considered to reduce the computational load without sacrificing model performance. Moreover, distributed computing frameworks and edge computing can facilitate more efficient training and inference by distributing computational tasks across multiple devices. Cloud-based platforms may also serve as a viable solution by providing scalable and on-demand computational resources, thereby enabling the deployment and testing of sophisticated models even in environments with limited local hardware capacity.

Study Limitations

Application of the SLR method in this research allowed the generalizability and consistency of research findings, following the goal to systematize and comprehend the scope of application of AI in the prediction of fabric properties and handfeel. To provide transparency, clarity, integration, focus, equality, accessibility, and coverage of the study, the authors strictly followed the PRISMA statement [13,55].

Despite these strengths, a few limitations should be noted. First, the study’s inclusion criteria focused on peer-reviewed databases and citation searches, which may have inadvertently excluded some relevant studies from other sources, potentially affecting the scope of the review. Second, the analysis was limited to English-language studies, which could introduce linguistic and retrieval biases. Third, no formal risk-of-bias assessment tool was applied, which might have influenced the study selection and interpretation. Nonetheless, independent reviews and consensus were conducted to minimize potential biases. Despite these constraints, the findings offer a robust basis for future research in AI-driven prediction of fabric properties.

### 5.2. Future Research Directions

Future research needs to address some of the identified challenges in this study by advancing AI and fabric handfeel. They can do so by using measuring and predicting technologies and their integration. Several promising directions are outlined below, focusing on improving model robustness, overcoming current limitations, and integrating cutting-edge AI techniques to propel innovation in textile science.

- Multidimensional Strategies for Optimizing Accuracy of AI Models

Future investigations should focus on enhancing the accuracy and generalizability of AI models by expanding the diversity and scale of training datasets. To overcome the limitations of existing datasets, which often lack adequate representation of various fabric properties, researchers can explore the use of synthetic data generation through Generative Adversarial Networks (GANs) or physics-based simulations. These techniques can create synthetic samples that emulate complex fabric behaviors, thereby increasing data diversity and mitigating the risk of overfitting.

Furthermore, transfer learning and meta-learning techniques can be employed to enable models to leverage knowledge from related tasks, reducing the need for extensive retraining when adapting to new fabric types or properties. This approach enhances the model’s ability to generalize across different textile applications. Additionally, the integration of hybrid modeling strategies, which combine linear regression, neural networks, and fuzzy logic, can further refine model robustness by capturing nonlinear relationships between fabric properties.

- Advanced Dynamic Simulation Techniques and Their Application in Innovative Clothing Design

Advancements in dynamic drape testing should emphasize the development of real-time simulation techniques to evaluate textile draping under various dynamic conditions, such as movement, stretch, and environmental changes. Future research could incorporate dynamic sensors and high-frame-rate imaging to capture deformation and movement data with high precision, which, when combined with deep learning models, can significantly enhance the realism and predictive accuracy of simulations.

Moreover, Reinforcement Learning (RL) can be explored to dynamically adjust simulation parameters based on real-time feedback, optimizing the model’s performance in various scenarios. This capability would allow researchers to simulate a wider range of fabric behaviors, providing deeper insights into how fabrics interact with different garment designs and movements, thereby supporting the development of innovative clothing solutions with enhanced ergonomic and aesthetic properties.

- Predicting Mechanical Properties of Fabrics Using Advanced Computational Models

The accurate prediction of mechanical properties such as tensile strength, bending stiffness, and shear force remains a complex challenge due to the intricate dependencies between different fabric attributes. Future research should explore the integration of neural networks with finite element analysis (FEA) or physics-based models to capture the fundamental mechanical interactions within fabrics. This hybrid approach would allow the development of models that can more accurately predict mechanical properties under various conditions.

To further improve the performance of these models, genetic algorithm-optimized neural networks (GA-ANNs) can be employed to fine-tune model parameters, ensuring robustness and reducing the risk of overfitting. Additionally, emerging AI techniques such as Transformer-based architectures can be explored for their capability to model complex interdependencies within fabric properties, enabling more accurate and comprehensive predictions.

- Enhancing Visual–Tactile Correlation Models in Textile Science

Accurately correlating visual and tactile perceptions remains a challenging task in fabric property prediction. Future research should focus on developing visual–tactile correlation models using advanced AI architectures such as Transformers and attention mechanisms. These models can capture long-range dependencies and complex interactions between visual attributes (e.g., texture, drape) and tactile properties (e.g., softness, elasticity), thereby improving the accuracy of tactile perception predictions.

In addition, future research can explore the use of neural-symbolic integration to combine deep learning with symbolic reasoning. This approach could enable models to incorporate domain knowledge about fabric mechanics and tactile perceptions, thereby enhancing model interpretability and providing more meaningful predictions for practical applications such as virtual fitting and online textile commerce.

- Advanced Pore Structure Analysis in Nonwoven Fabrics

Future research should aim to enhance the precision of pore structure analysis in nonwoven fabrics by integrating high-resolution imaging techniques (e.g., computed tomography and electron microscopy) with AI-driven optimization. This integration would allow for a more detailed assessment of pore configurations and their influence on properties such as thermal permeability and wear comfort.

Moreover, employing Generative Adversarial Networks (GANs) or deep neural networks for modeling and optimizing pore structures could provide new insights into fabric design, enabling the development of nonwoven textiles with tailored attributes that meet specific application requirements. This would significantly contribute to the advancement of next-generation textiles with enhanced functionality and sustainability.

## 6. Conclusions

This systematic review identifies and categorizes the most effective AI models and techniques for predicting various fabric properties, such as mechanical characteristics and tactile perception. AI techniques, including extreme learning machines, genetic algorithms, fuzzy logic, adaptive neuro-fuzzy inference systems (ANFISs), and deep learning, have demonstrated high accuracy and adaptability across different fabric types, making them suitable for real-world applications.

For tactile qualities like softness, smoothness, and flexibility, methods such as fuzzy logic and ANFISs have shown outstanding performance, achieving up to 95% accuracy. Deep learning models like ResNet-50 have demonstrated even greater precision, reaching 99.3% accuracy in classifying woven fabric textures. These results highlight their robustness in managing complex visual and tactile data.

For mechanical properties, including tensile strength, elasticity, and bending stiffness, AI models like artificial neural networks (ANNs), convolutional neural networks (CNNs), and finite element analysis (FEA)-based hybrid models have shown exceptional predictive capabilities. For example, ANN models have yielded low root mean square errors (RMSE) in predicting tensile strength, while CNNs have effectively predicted bending stiffness with error margins below 10%.

In the context of fabric drape, AI techniques such as fuzzy logic and CNNs with self-attention mechanisms have demonstrated high correlation coefficients and strong prediction accuracy. These models can simulate fabric movement and drape behavior with error rates as low as 1%, making them highly effective in replicating results from traditional physical testing methods.

Overall, this review confirms that hybrid models combining machine learning with physics-based simulations and advanced deep learning architectures are among the most promising approaches for future research. These models are well-suited for handling complex, multidimensional datasets and scalable prediction scenarios. Further developments in data generation techniques, such as Generative Adversarial Networks (GANs), and advanced architectures like Transformers will enhance the robustness and generalization capabilities of these models.

Practical applications of AI models, such as using fuzzy logic for predicting fabric saturation levels, have already demonstrated real-world benefits by achieving a mean absolute error of only 1.97%, reducing weaving issues and material waste. AI-powered digitalization for 3D garment prototyping has streamlined design processes, reduced reliance on physical measurements, and significantly improved cost efficiency and productivity. Additionally, neural networks have effectively predicted the degradation of mechanical properties in aramid fabrics, enhancing durability and safety in protective clothing. An Intelligent Decision Support System (IDSS) utilizing large-scale data has also achieved high accuracy in predicting elasticity and pilling, reducing the time and cost of fabric prototyping and optimizing textile design.

These successful applications illustrate AI’s transformative potential in advancing sustainable textile practices and optimizing fabric property prediction. However, challenges such as limited data generalizability and high computational requirements persist. Future research should focus on overcoming these challenges through advanced methods like synthetic data generation and hybrid modeling, which will further enhance model robustness and adaptability, ultimately paving the way for more sustainable, efficient, and innovative textile manufacturing processes.

This review was not registered in any formal database or review registry.

A formal review protocol was not prepared for this systematic review. The study followed PRISMA guidelines but did not have a pre-established protocol.

Since the review was not registered and no protocol was prepared, no amendments were made to any registration or protocol during the study.

## Figures and Tables

**Table 1:** Study selection criteria.

Inclusion Criterion	Value
Papers related to the textile industry	Include
Papers written in the English language	Include
Title includes at least one searched keyword	Include
Abstract includes at least one searched keyword from each topic	Include
Abstract is relevant to the research question	Include
Articles that lacked detailed descriptions of AI models, algorithms, or datasets, or had insufficient full-text access for analysis	Exclude
Studies focusing solely on textile processing rather than fabric property prediction	Exclude
studies that demonstrated high levels of selection bias, performance bias, or reporting bias	Exclude

**Table 2:** Summary of key studies of AI techniques for predicting fabric handfeel characteristics.

Fabric Properties	Predictive Features	AI & Techniques	Dataset Size	Accuracy Rate	Reference
**Texture recognition** (**roughness, smoothness**)	Spatiotemporal spike patterns derived from tactile sensors	Extreme learning machine	10 graded textures with 50 data samples	92% classification accuracy	Rasouli et al. [40]
**Tactile properties** (**softness, smoothness, fullness, flexibility, delicacy, lightness, resiliency**)	Relations between tactile properties and total preference for men’s suits	Genetic algorithm, fuzzy comprehensive evaluation	50 textile fabrics with various tactile properties	Close to genetic algorithm solution: 95% (accuracy in weight distribution)	Xue et al. [41]
**Tactile properties** (**softness, smoothness, flexibility, etc.**)	Visual features (drape, fit at abdomen and hip, wave size, etc.)	ANFISs	18 textile samples used for training; 3 additional samples for testing	Predictive errors for tactile properties do not exceed 1 on an 11-point scale	Xue et al. [42]
**Tactile properties**	Visual features	Deep learning, ResNet-50	11,328 images (training), 2832 images (testing)	99.3% accuracy in woven fabric types; error level below 10% in yarn quality evaluation	Gültekin et al. [43]
**Mechanical properties** (**tensile, shearing, bending, compression, surface friction**)	Total hand value and tactile comfort scores predicted by using low-stress mechanical properties	Artificial neural networks, ANFISs	486 measurements: 15 mechanical properties * 6 samples * 3 replicas * 2 directions	ANN RMSE: 0.014; ANFIS RMSE: 0.0122, with significantly lower errors than standard deviations (ANN: 0.644, ANFIS: 0.85)	Tadesse et al. [29]
**Tactile comfort** (**warm–cool, itchy–silky, etc.**)	Hand value and total hand value predicted from sensory attributes	Fuzzy logic model, ANN	9 functional fabrics, various finishing parameters	FLM RMSE: 0.21; ANN RMSE: 0.13; FLM RMPE < 10%; ANN RMPE: 2.24%	Tadesse et al. [28]

**Table 3:** Summary of key studies of AI techniques for predicting mechanical properties of fabric.

Fabric Properties	Predictive Features	AI & Techniques	Dataset Size	Rate of Accuracy	Reference
**Fabric Shear and Deformation**	Shear force, deformation patterns, normal stress, von Mises stress	Finite element analysis, Bernoulli–Euler beam theory, Coulomb’s friction model	Multiple shear angles simulated; detailed yarn and fabric unit cells analyzed	Good agreement between finite element analysis and theoretical predictions, accuracy rate not explicitly stated but shown in comparisons	Basit and Luo [44]
**Fabric Strength**	Bursting Strength, Tensile Strength	Neural network, regression models	20 fabric samples	R^2^ = 0.765 (regression model), fuzzy logic close to real values	Kilic [45]
**Elastic Properties**	Warp and weft elasticity, Bias distortion, Pilling prediction from fabric design features	Automated machine learning, multi-target regression using deep artificial neural network	8650 fabric examples	NMAE: 4% for weft elasticity, 11% for pilling, 87% accuracy for textile composition	Ribeiro et al. [46]
**Air permeability, Porosity**	Fiber distribution, areal weight, texture features	Artificial neural networks	192 image frames	High regression (R = 0.99 for air permeability)	Gültekin et al. [43]
**In-Plane Shear Properties**	Shear force, deformation under bias-extension test	Finite element analysis, analytical methods	Various textile composite reinforcements and prepregs	Agreement between experimental and simulated shear behavior	Boisse et al. [47]
**Bending Stiffness**	Multi-view depth images of draped fabric specimens	Deep neural networks, Simulation-in-the-loop	618 real-world fabrics; 2.3 M synthetic depth images	Improved simulation fidelity; exact accuracy rate not stated but significant improvement over traditional methods	Feng et al. [23]
**3D Textile Architecture**	Yarn paths, weave initial architecture	Convolutional neural networks, long short-term memory	4000 weaving architectures	Stiffness properties prediction error < 10%	Koptelov et al. [48]
**Yarn-Level Fabric Mechanics**	Stiffness, Nonlinearity, Anisotropy of knitted fabrics	Yarn-level simulation, thin-shell model, parameter fitting	33 different knitted fabrics	Avg. error: 17.59% ± 8.33% for stretch force, 16.84% ± 8.11% for compression	Sperl et al. [49]
**Mechanical Properties of Woven Composites**	Fiber angles, resin material parameters, and effective modulus	Convolutional neural networks, finite element analysis	3000 woven fiber composites	Average error < 5% compared to FEM results	Hsu et al. [50]
**Textile Polymer Composite Materials**	Tensile strength, compressive strength, bending strength, elongation at break	Multi-objective optimization, neural networks, support vector machines	420 samples with 11 physical characteristics	Optimized ANN accuracy: 90.2%; SVM accuracy: 89.9%	Malashin et al. [51]

**Table 4:** Summary of key studies of AI techniques for predicting fabric drape.

Fabric Properties	Predictive Features	AI and Techniques	Dataset Size	Accuracy Rate	Reference
Fabric Drape	Drape coefficient, flexural rigidity, tensile elongation	Fuzzy logic, image analysis	20 fabric samples	Fuzzy logic method provides results close to those with Cusick’s method (accuracy within 1%)	Kilic [45]
Drape Behavior of 3D Woven Fabrics	Shear force, tensile, and bending behavior of 3D woven fabrics	FEM, shell elements, hyperplastic model	Various textile composite reinforcements	Validation against experimental results with good agreement	Hübner et al. [52]
Fabric Drape Behavior	Drape coefficient, node number, drape distance ratio, fold depth index	Fuzzy logic	63 woven fabrics	High correlation: DC (0.943), NN (0.936), DDR (0.969), FDI (0.946)	Hamdi et al. [53]
Garment Fit and Drape Characteristics	Fit score evaluation based on body dimensions and drape simulation	ANN, drape simulation	15–17 sizes for training, multiple body models	Not explicitly stated, but improved fit scores compared to traditional methods	Oh and Kim [54]
Fabric Drape and Mechanical Properties	Drape coefficient, stretch stiffness, bending stiffness	CNN with ResNet-18 and self-attention mechanisms	8 fabric samples (5 knit, 3 woven)	NMAE for AI-based drape: 3–51%; for PT-based drape: 2–11%	Youn et al. [6]
Drapability and Tactile Sensation (Softness)	Drape coefficient, softness	Fuzzy C-means clustering, ANN	777 fabric samples	ANN prediction accuracy: 83.5%	Lee et al. [20]

## Data Availability

Not applicable.

## Abbreviations

Fabric Drape	Drape coefficient, flexural rigidity, tensile elongation
AI	Artificial Intelligence
ANN	Artificial Neural Network
ANFIS	Adaptive Neuro-Fuzzy Inference System
CNN	Convolutional Neural Network
FEM	Finite Element Method
GAN	Generative Adversarial Network
IDSS	Intelligent Decision Support System
KES	Kawabata Evaluation System
MAE	Mean Absolute Error
NMAE	Normalized Mean Absolute Error
RMSE	Root Mean Square Error
SLR	Systematic Literature Review
RL	Reinforcement Learning
STR	Single-Target Regression
RF	Random Forest
PCA	Principal Component Analysis
GA	Genetic Algorithm
FAST	Fabric Assurance by Simple Testing
R-CNN	Region-based Convolutional Neural Network
